# Preclinical Evidence for the Benefits of Penile Rehabilitation Therapy following Nerve-Sparing Radical Prostatectomy

**DOI:** 10.1155/2008/594868

**Published:** 2008-06-25

**Authors:** M. Albersen, S. Joniau, H. Claes, H. Van Poppel

**Affiliations:** Department of Urology, University Hospitals Leuven, 3000 Leuven, Belgium

## Abstract

Erectile dysfunction following radical prostatectomy remains a frequent problem despite the development of nerve-sparing techniques. This erectile dysfunction is believed to be neurogenic, enhanced by hypoxia-induced structural changes which result in additional veno-occlusive dysfunction. Recently, daily use of intracavernous vasoactive substances and oral use of PDE5-inhibitors have been clinically studied for treatment of postprostatectomy erectile dysfunction. Since these studies showed benefits of “penile rehabilitation therapy,” these effects have been studied in a preclinical setting. We reviewed experimental literature on erectile tissue preserving and neuroregenerative treatment strategies, and found that preservation of the erectile tissue by the use of intracavernous nitric oxide donors or vasoactive substances, oral PDE5-inhibitors, and hyperbaric oxygen therapy improved erectile function by antifibrotic effects and preservation of smooth muscle. Furthermore, neuroregenerative strategies using neuroimmunophilin ligands, neurotrophins, growth factors, and stem cell therapy show improved erectile function by preservation of NOS-containing nerve fibers.

## 1. INTRODUCTION

Cancer of the prostate is now recognized as one of the principal medical problems in the male population [1]. At present, radical prostatectomy (RP), either retropubic, perineal, laparoscopic, or robot-assisted laparoscopic, is the treatment of choice in young men with clinically localised prostate cancer. Since prostate cancer is detected at increasingly younger age and lower stage, patients undergoing radical prostatectomy generally have good baseline erectile function, and have high expectations concerning the preservation of erectile function following the procedure. Since Walsh and Donker published their insights in the etiology and prevention of impotence following retropubic RP in 1982, the nerve-sparing technique they described is widely employed to improve postoperative erectile function [2, 3]. Literature research reveals widely disparate potency rates between various studies concerning nerve-sparing RP (6–86%) [4–12]. Despite the development of new techniques for preservation of the cavernous nerves, many men continue to suffer from erectile dysfunction (ED) and penile shortening after RP, due to neuropraxia. In 1997, Montorsi and colleagues introduced the concept of early postoperative vasoactive therapy and penile rehabilitation, and they suggested that the early postoperative use of intracavernosal injection therapy to promote penile erection may result in improved erectile function outcomes [13]. More recently, PDE-5-inhibitors have been studied for their use in penile rehabilitation [14, 15]. Good results have been achieved with the use of nerve grafts in nonnerve sparing surgery, and the use of neuroregenerative tubular implants [16, 17]. This review article attempts to summarize the contemporary basic scientific knowledge on the pathophysiological mechanisms of post-RP ED and to review current basic science evidence for medicinal, nonchirurgical penile rehabilitation therapy and neuroregenerative therapies.

## 2. ANATOMY AND PHYSIOLOGY OF THE ERECTION

The penile corpora cavernosa and the corpus spongiosum are innervated by the combined sympathetic and parasympathetic cavernous nerves (CNs), which arise from the pelvic plexus. These nerves are condensed in the neurovascular bundles (NVBs), which can be found in close relation to the dorsolateral side of the prostatic capsula and urethra, although recent findings suggest that a significant number of nerves can be found along the ventral parts of the prostatic capsula (i.e., “outside” the classic NVB) [18–20]. Nitric oxide (NO), released during nonadrenergic, noncholinergic (NANC) stimulation from the CN terminals and from the endothelium is the principal neurotransmitter mediating penile erection. NO activates guanylyl cyclase, an enzyme that raises the intracellular concentration of cyclic guanosine monophosphate (cGMP), leading to activation of cGMP specific protein kinases which activate further intracellular events, eventually leading to reduction of intracellular calcium, and relaxation of the smooth muscle. cGMP is hydrolysedto GMP by phosphodiesterase type 5 during return to the flaccid state. The vasodilator prostaglandin E1 (PGE1) also causes SM relaxation but by increasing the concentration of the cyclic adenosine monophosphate (cAMP), via stimulation of adenylate cyclase. The resulting vasodilation results in an increase in penile blood flow. During this phase of tumescence, relaxation of the trabecular smooth muscle increases the compliance of the sinusoids, resulting in expansion of the sinusoidal system. The subtunical venous plexusesare thus compressed between the expanding sinusoidal wall and the noncompliant tunica albuginea, resulting in almost total subtunical venous occlusion. These events, augmented by contraction of the ischiocavernosus muscles, trap the blood within the corpora cavernosa, with an intracavernous pressure that can approach several hundreds of mm Hg [21–24].

## 3. PATHOPHYSIOLOGIC MECHANISMS OF ERECTILE DYSFUNCTION FOLLOWING RADICAL PROSTATECTOMY

### 3.1. Impairment in corpus cavernosum oxygenation

#### 3.1.1. Physiological changes in penile oxygen tension

Azadzoi and colleagues showed in a canine model that subtunical oxygen tension in the *flaccid* penis was close to 100 mmHg, consistent with a largely arterial circulation, whereas deep cavernosal oxygen tension measurements showed an oxygen tension consistent with venous blood flow. With pelvic nerve stimulation or injection of vasoactive agents, oxygen tension deep within the corpus cavernosum increased during *penile erection* from a level consistent with venous blood to a level consistent with arterial blood. Also, due to the increase of intracavernosal pressure, blood flow in the subtunical blood flow significantly decreased [25]. Various investigators state that a persistent flaccid state of the penis, as seen after cavernous neurotomy with absence of nocturnal erections, causes a relatively hypoxic state causing a variety of structural and functional changes in the corpora cavernosa.

#### 3.1.2. Nocturnal penile tumescence (NPT)

Nocturnal penile tumescence is a sleep-related phenomenon that occurs during the rapid eye movement sleep-phase in men without erectile dysfunction. These nocturnal erections occur 3–5 times per night, lasting 30–45 minutes [26]. During these phases of NPT, arterial oxygen is supplied to the cavernous tissue. This supply of oxygen is necessary for the formation of NO by nitric oxide synthase (NOS) in the cavernosal endothelium (eNOS) and in the CN endings (nNOS). Upon its release, NO diffuses locally into smooth muscle cells and binds with its physiologic receptor, soluble guanylyl cyclase, which catalyses the conversion of GTP to cGMP. This second messenger eventually evokes a decline in cytosolic calcium resulting in smooth muscle relaxation and penile tumescence [20, 24]. Bannowsky et al. showed absence of NPT in a control group without nerve-sparing versus 97% NPT in the group that underwent nerve sparing RP [27]. This absence of NPT following cavernous neurotomy causes a permanent lack of free oxygen in the corpus cavernosum, resulting in a predisposition to cavernosal fibrosis [19, 28]. In a rat model, this neurotomy-induced hypoxia was demonstated by significantly higher protein expression and immunohistochemical staining of hypoxia-inducible factor-1*α* (HIF-1*α*) in the neurotomised rat [28]. HIF-1*α* is a transcription factor that is expressed when mammalian cells are subjected to hypoxia. This cytokine activates the transcription of genes encoding proteins that are important for maintaining oxygen homeostasis. This finding confirms the theory that hypoxia of rat penes after iatrogenic nerve injury was induced by the loss of nocturnal erections [29].

#### 3.1.3. Altered contractility of penile smooth muscle by penile oxygenation

Various investigators have shown that responses of blood vessels to NO were inhibited at low oxygen tensions [30–34]. Kim et al. have studied this effect of oxygen tension on the resistance of the arterial bed of the penis and on the relaxation of cavernosal trabecular smooth muscle. They showed that isolated human and rabbit corpus cavernosum tissue strips relaxed after addition of endothelium-dependent dilator acetylcholine and to electrical stimulation of the autonomic nerves, when bathed in an organ-bath with arterial-like PO_2_. These (NO-mediated) responses progressively were inhibited when PO_2_ levels where decreased to levels measured in the deep corpus cavernosum of the flaccid penis. Restoring PO_2_ levels to normoxic conditions restored this endothelium-dependent and neurogenic relaxation. Furthermore, they showed that low PO_2_ reduced basal levels of cGMP, prevented cGMP accumulation induced by nerve stimulation, and inhibited cytosolic NOS activity. They concluded that penile concentration of oxygen modulates penile erection by regulating NO synthesis in the corpus cavernosum [35].

### 3.2. Structural changes following cavernous neurotomy

A number of patients who have undergone radical prostatectomy complain of subjective penile shortening. Various clinical studies have addressed this finding, with a reported incidence of penile shortening of in 9–71% of patients [36–39]. User et al. showed a loss in penile wet weight in a rat model following bilateral neurotomy, although unilateral neurotomy showed a much greater preservation of penile weight. The majority of authors suggest that penile fibrosis accounts for these morphometric changes, by reducing the extensibility and volume of the corpus cavernosum [40]. Also, denervation-induced apoptosis has been proposed to play a role in penile shortening after neurotomy [41]. Mulhall recently proposed a hypothesis that penile size reduction should be divided into early postoperative and reversible size reduction (hypothised to be due to postoperative sympathetic overdrive), and delayed and permanent structural changes due to hypoxia and denervation-induced structural changes [42].

#### 3.2.1. Postoperative early reversible penile size reduction

The hypothesis of Mulhall that early penile size reduction is the result of sympathetic hyperinnervation is based on the fact that nerve growth factor (NGF), a neurotrophin which is released after peripheral nerve damage, induces differentiation of sympathetic neurons and enhances target innervation [43, 44]. This mechanism is suspected to induce sympathetic hyperinnervation of the corpus cavernosum, following an earlier regeneration of sympathetic nerve fibers, resulting in smooth muscle contraction and a hypertonic state of the penis. This hypothesis was supported by a recently published clinical study of Gontero and colleagues [39].

#### 3.2.2. Postoperative permanent structural changes: apoptosis

Klein et al. stated that, given the observation that penile size may decrease after radical prostatectomy, damage to the cavernous nerves might result in apoptosis of cells in erectile tissues of the penis. They found the presence of condensed and fragmented cell nuclei (characteristic of apoptotic cells) within the erectile tissue, and expression of sulfated glycoprotein-2 (a protein found to increase dramatically in several urogenital tract tissues undergoing apoptosis), in denervated rat-penises versus a sham-operated control group [41]. User and colleagues have elucidated the role of apoptosis in the pathophysiology of postprostatectomy ED in a postpubertal male Sprague-Dawley rat model. Rats were randomized to bilateral or unilateral CN transection versus a sham operation. At different time intervals following the procedure, penile wet weight, DNA content, and protein content were measured. Tissue sections of the penis were stained for apoptosis, and the apoptotic index was calculated. Finally, staining for endothelial and smooth muscle cells was done to identify the apoptotic cell line. They found that bilateral cavernous neurotomy induced significant apoptosis. In addition, it was found that most apoptotic cells were located just beneath the tunica albuginea where the subtunical venular plexus is located. Finally the authors demonstrated that apoptotic cells were predominantly smooth muscle cells and not endothelial cells [40, 45]. This finding strongly implies that smooth muscle cells somehow depend on neural innervation. The programmed cell-death of smooth muscle has further implications. It has been shown that reductions in the quantity of smooth muscle cells is a pivotal event in the development of veno-occlusive dysfunction, which has been shown to play a major role in postprostatectomy ED [46, 47].

Sonic hedgehog homolog (SHH) is one of three proteins in the mammalian hedgehog family. It plays a key role in regulating vertebrate organogenesis and remains important in the adult. It controls cell division of adult stem cells. SHH was suggested to play a role in apoptosis of smooth muscle cells. In a series of experiments by Podlascek et al., bilateral CN resection resulted in decreased SHH in the smooth muscle of the corpora cavernosal sinusoids and extensive morphological changes, including increased apoptosis. The SHH cascade acts to establish normal penile morphology. Nerve injury disrupts the SHH cascade and corpora cavernosal homeostasis such that morphological changes in sinusoid structure ensue, resulting in ED. Bond et al. illustrated that neural activity and a trophic factor from the pelvic ganglia or cavernous nerves are necessary to regulate SHH protein and smooth muscle abundance in the penis [48–51].

#### 3.2.3. Postoperative permanent structural changes: fibrosis

Leungwattanakij and associates evaluated the isolated effects of CN injury on cavernous oxygenation and cavernous fibrosis using the Sprague-Dawley rat. They showed that production of transforming growth factor-*β*_1_ (TGF-*β*_1_) and TGF-*β*_1_ mRNA expression as well as collagen I and III protein expressions was significantly greater in the cavernous neurotomy-group compared to a sham-operated group [28]. TGF-*β*_1_ is associated with increased collagen synthesis by cavernous smooth muscle cells, resulting in cavernosal fibrosis. Furthermore, TGF-*β*_1_ dependent endothelin-1 (ET-1) synthesis is increased. ET-1 is a potent constrictor of penile smooth muscle and a profibrotic peptide [52]. Studies also showed that low oxygen tension in human cavernosal tissue inhibits production of PGE1. PGE1 inhibits collagen formation by inhibiting TGF-*β*_1_ that induces collagen synthesis. With the inhibition of PGE1, TGF-*β*_1_ is allowed to induce connective tissue synthesis [53–59]. Prolonged periods of cavernosal hypoxia thus lead to increased deposition of connective tissue that may finally lead to a decrease in penile distensibility, veno-occlusive dysfunction, ED, and reduction in penile length.

## 4. CORPUS CAVERNOSUM OXYGENATION PRESERVING STRATEGIES

### 4.1. Intracavernous injections of vasoactive agents

The finding that poor cavernosal oxygenation induces cavernosal structural damage which causes erectile dysfunction following radical prostatectomy leads to the hypothesis that preserving the oxygenation of the corpora cavernosa by improving blood flow might improve erectile dysfunction. Montorsi et al. conducted a pioneering clinical study on the effects of intracavernosal injections of aldoprostil early after bilateral nerve sparing prostatectomy and saw a significantly higher recovery of spontaneous erections in treated patients treated versus observation alone [13].

Preclinical data on the use of intracavernosal and intraurethral vasoactive agents is limited. Moreland et al. found that in vitro TGF-*β*_1_ mRNA induction was suppressed by PGE1 via increased levels of cAMP. These data suggest that prostaglandins and TGF-*β*_1_ may play a key role in modulation of collagen synthesis in the corpus cavernosum, and in the regulation of fibrosis of the corpus cavernosum [55–60]. A cross-regulation of intracellular cGMP and cAMP in cultured human corpus cavernosum smooth muscle cells was found by Kim et al. They showed a rise in cGMP (which has been identified as an antifibrotic mediator) as a response to a rise in intracellular cAMP, which is accomplished by intracavernous injection of PGE1 [61, 62]. These in vitro observations are likely to play a major role in the recovery of spontaneous erections in patients treated with intracavernous PGE1 following nerve sparing radical prostatectomy.

### 4.2. PDE-5-inhibitors (PDE5-I)

PDE5-I act within the smooth muscle cells where the enzyme phosphodiesterase-5, an enzyme which naturally degrades cGMP, is inhibited. This causes increased levels of cGMP, leading to activation of cGMP specific protein kinases which activate further intracellular events, eventually leading to reduction of intracellular calcium, being associated with cavernosal smooth muscle relaxation. The mechanism of PDE5-I is thus dependent on the production of NO from the nerve endings, which initiates production of cGMP.

Padma-Nathan and colleagues reported the results of a randomized, placebo-controlled study examining the benefits of nightly administration of sildenafil during the postoperative period following a bilateral nerve-sparing RRP. This study included 76 men with normal preoperative erectile function. Forty-eight weeks after bilateral nerve-sparing RRP, 27% of patients receiving sildenafil demonstrated return of spontaneous erectile function, compared with 4% in the placebo group. Postoperative NPT assessments supported these patient-reported results [15]. Since Padma-Nathan et al. published their results of the clinical applicability of PDE5-I, preclinical effects of this class of drugs have been the focus of intense investigation.

Ferrini et al. were the first to demonstrate that long-term continuous oral administration of a PDE5-I (vardenafil) in rats prevents neuropraxia-induced veno-occlusive dysfunction, presumably as a result of the antifibrotic effects of PDE5-I and the preservation of the corporal SM mass by upregulation of SM cell replication [63]. The same group demonstrated the same antifibrotic effects and prevention of veno-occlusive dysfunction with the use of the other PDE5-inhibitors, sildenafil and tadalafil, and showed that these effects where cGMP mediated, but independent of inducible NOS (iNOS) upregulation, as they hypothesised in their vardenafil experiment [62–64]. Vignozzi et al. demonstrated in vitro that bilateral CN neurotomy induced penile hypoxia shown by a dramatically increased hypoxyprobe labeling, a generally accepted probe of hypo-oxygenation after a 3-month interval from surgical neurotomy. They found that chronic tadalafil treatment almost completely restored penile oxygenation and smooth muscle/fibrous tissue ratio at histology [65]. They also found that sildanefil administration was able to counteract penile hypoxia, expression of profibrotic endothelin-1 type B receptor (ETB), and hypersensitivity to a ETB agonist IRL-1620 (using an in vitro contractility study), with its effect being more evident the earlier it was administered [66]. These studies indicate that PDE5-I can potentially protect and preserve the integrity of the corpora after cavernosal nerve damage when given on a long term and continuous basis [67].

At genomic level, the impact of sildanefil treatment on gene expression in the rat CN injury model was evaluated by Müller et al. of the Memorial Sloan-Kettering Cancer Centre in New York. They used microarray analysis to elucidate which genes are altered by administration of PDE5-I in the CN injury model and identified genes that are altered in the CN injury model and that are restored towards normal levels with the use of sildenafil. These genes predominantly belonged to apoptosis, neurotrophism, and endothelial funtion families [68].

### 4.3. Hyperbaric oxygen therapy

Hyperbaric oxygen therapy (HBOT) has been used in the treatment of a variety of medical conditions, in the urology field especially for radiation-induced cystitis. Oxygen hypersaturation has been proven to induce angiogenesis and enhance wound healing based on the excessive supply of oxygen. Since hypoxia is assumed to be the capital factor in inducing penile structural changes and erectile dysfunction following cavernous nerve injury, Müller et al. [69] investigated the effects of hyperbaric oxygen therapy, inducing hyperoxygenation in this setting. They used a bilateral cavernous nerve crush-injury Sprague-Dawley rat model which was exposed to ten sessions of hyperbaric oxygen at three atmospheres for the duration 90 minutes. They measured ICP after CN stimulation under controlled conditions with continuous systemic blood pressure measurement, which according to the authors provides an accurate measurement of erectile response. They found that rats in the HBOT group had significantly higher mean ICP/MAP ratios compared with the untreated group. They demonstrated preservation of smooth muscle/collagen ratio with HBOT using Masson’s Trichrome coloring of penile cross-sections. This study was the first to show a protective effect of HBOT on erectile function, and literature data is therefore limited [69]. Theoretically, induction of angiogenesis by HBOT might potentially accelerate tumor growth in cancer patients and could therefore be unfavourable in patients treated for pelvic malignancies. This issue was however studied in a mouse model that was developed to investigate the effect of HBOT on prostate cancer cell growth in patients treated for radiation-induced hemorrhagic cystitis. The investigators did not witness increased rates of tumor growth, and they concluded that HBOT in cases of prostate cancer is not associated with increased oncological risk [70].

### 4.4. Nitric oxide donor therapy

Since neuropraxia causes loss of NOS containing nerves and hypoxia, resulting in a decrease in available NO and structural changes, Veliev and associates hypothesised that direct restoration of NO in the penis could prevent these changes after denervation. This group investigated the effect of intracorporeal administration of new NO donor dinitrosyl iron complex in a bilateral CN crush injury group compared to a sham-operated group and an untreated group CN crush injury group. They used analysis of mitotic activity of smooth muscle (decreased after CN injury), endothelium, and fibroblasts (both increased after CN injury) for evaluation of cavernous fibrosis development and found a stabilisation of fibroblast proliferation and improvement of smooth muscle mitotic activity in the group treated with intracavernosal injections of NO donor [71].

## 5. NEUROMODULATORY STRATEGIES

During radical prostatectomy, even with the most advanced nerve-sparing dissection techniques, neuropraxia, stretching, and axotomy of the cavernous nerves are inevitable. Although the peripheral nervous system is capable of endogenous regeneration, this response is limited and usually does not allow full recovery of nerve function. Cavernous nerves have been shown to possess the capability to regenerate in 6 months. Recently, accumulating evidence suggests that a return of potency following radical prostatectomy is partially dependent on regeneration of the cavernous nerves and axonal regeneration in the remaining neural tissue. Several treatment strategies to improve this therapeutic neurogenesis have been under investigation in animal models, including immunophilin ligands, neurotrophins, growth factors and, more recently, stem cell therapy [45, 72].

### 5.1. Immunophilin ligands

Immunophilin ligands *cyclosporin A* and *FK506* (Tacrolimus) are short polypeptides which block the activation of lymphocytes and other immune cells. In cells, FK506 and cyclosporine A bind to intracellular proteins called immunophilins. The formed complex inhibits the activity of calcineurin, a cellular signaling protein which is one of the key enzymes regulating the activity of many proteins in neurons [71]. Immunophilin ligands have been shown to hold neuroprotective and neuroregenerative potential in the central nervous system as well as in peripheral nerves. FK506 showed both functional recovery and nerve regeneration in the sciatic nerve after crush injury in an animal model [73, 74]. This success brought immunophilin ligands under interest for the protection of penile innervation following CN injury. Administration of FK506 orally or intraperitoneally was found to protect erectile function measured by intracavernous pressure monitoring after electrical stimulation of the CN following CN injury. Immunohistochemical evaluation of a CN crush model treated with FK506 showed maintained peripheral unmyelinated axon integrity and CN architecture in a bilateral nerve crush injury rat model. The crush injury was used to simulate the effects of nerve-sparing radical prostatectomy, where neuropraxia occurs more likely than neurotomy [75, 76]. FK506 also induced high glutathione peroxidase expression, an antioxidant enzyme, suggesting that FK506 promotes erectile recovery through antioxidative mechanisms [77].

### 5.2. Nonimmunosuppressant lmmunophilin ligands

There are some concerns about the application of immunosuppressant (and carcinogenic) qualities of FK506 therapy in prostate cancer patients, although daily administration of a dose of 2-3 mg/day in a study by Fleischmann et al. in humans with rheumatoid arthritis did not induce immunosuppression [78]. Therefore, the use of nonimmunosuppressant immunophilin ligands has recently gained interest of researchers. Nonimmunosuppressant molecules have recently been developed: *GPI1046* by (Guilford Pharmaceuticals Inc., Md, USA) (now MGI Pharma), *FK1706* by (Astellas Pharmaceuticals Inc., Ill, USA). Valentine et al. studied the effects of GPI1046 administration on CN integrity and erectile function under various conditions of CN crush injury. They found that oral or intraperitoneal administration of GPI1046 preserved intracavernous pressure (ICP) after electrical CN stimulation, compared to vehicle treated animals. They also evaluated ultrastructural integrity of the distal CN and found preservation of unmyelinated axons compared to the vehicle-treated group [79]. GPI1406 was shown not to promote prostate cancer cells in vitro [80]. Bella et al. proved that subcutaneous administration of FK1706 was effective in enhancing the recovery of erectile function in a concentration-dependent manner. With a high-dose treatment group showing significantly increased ICP compared to the vehicle-treated group in response to electrical CN stimulation. Sham treated animals showed regular axon sizes and shapes with homogenous GAP-43 and neurofilament staining, whereas injured axons showed irregular shapes, sizes, and staining patterns. FK1706 treatment restored axon shape and staining patterns. Injury significantly decreased nicotinamide adenine dinucleotide phosphate staining and FK1706 treatment showed a trend toward increased staining [81, 82].

### 5.3. Neurotrophins and growth factors

The effect of growth factors on erectile recovery and nerve regeneration has been extensively studied by Lue’s group at the Knuppe Laboratory for Molecular Urology in San Fransisco. They previously reported enhanced NOS-containing nerve fiber regeneration after injury, using injections of systemic growth hormone (GH) and insulin-like growth factor-1 (IGF-1). However, some obvious concerns are existing about the systemic use of growth factors in patients treated for malignancy.

#### 5.3.1. Brain derived neurotrophic factor

Neurotrophins are proteins that enhance neuronal regeneration and survival of specific neuronal populations through a variety of signaling pathways [77]. Brain derived neurotrophic factor (BDNF) has been the focus of intense investigation because of the central role it plays in neuronal development, maturation, and survival after injury [72]. It is part of the family of growth factors that includes NGF and neurotrophin-3, -4, and -5. It has demonstrable retrograde axonal transport, which occurs as molecules that are taken up by the neural synapses at the corpus cavernosum and travel retrograde to the cell body in the major pelvic ganglion to induce their neuroregenerative effects. This characteristic of BDNF makes it a suitable molecule for intracavernous injection. Lue’s group demonstrated that intracavernous injection of adeno-associated virus-BDNF prevents the degeneration of neuronal NOS containing neurons in the major pelvic ganglion and facilitated regeneration of NOS containing nerve fibers in penile tissue, thus enhancing erectile recovery following bilateral CN injury. They identified the JAK/STAT pathway as primary mechanism for in vitro BDNF-mediated neurite outgrowth. They also demonstrated a synergistic effect of BDNF when combined with vascular endothelial growth factor (VEGF) on neural and erectile recovery [83].

#### 5.3.2. Glial cell-line derived neurotrophic factors

Glial cell-line derived neurotrophic factors (GDNFs) are a novel group of neuroregenerative molecules of which the most studied are GDNF and neurturin (NRTN). Studies performed by Leitner et al. and Laurikainen et al. showed retrograde axonal transport of NRTN when injected in the penile shaft and suggested an effect of NRTN as a target-derived neuritogenic factor for postganglionic neurons via a system mediated by the GDNF receptor family, mainly the GDNF-*α*2 receptor [72, 84, 85]. In vivo, NRTN was able to preserve erectile function 5 weeks after nerve crush injury when applied to the site of nerve injury compared to a control group where albumin was applied locally, with an ICP increase of 55% compared to the control group [86].

#### 5.3.3. Growth differentiation factor 5

Growth differentiation factor-5 (GDF-5) is a recently isolated neurotrophic factor which belongs to the TGF-*β* superfamily, which was previously proven to be a neurotrophic and neuroprotective factor on dopaminergic neurons after intracerebral injection in vitro and in vivo. Data concerning the peripheral nervous system is limited to sensory neurons. Fandel et al. [87] investigated the in vivo effects of implantation of a GDF-5 impregnated sponge in the corpus cavernosum of a rat CN crush injury model. They found that intracavernosal application of GDF-5 enhanced the recovery of erectile function and preservation of n-NOS containing nerve fibers, with a low-dose application giving the most promising results [87].

#### 5.3.4. Erythropoietin

Erythropoietin (Epo) was originally known for the erythropoiesis. It is has now become evident that Epo is a multifunctional trophic factor. Epo for example has potent neurotrophic activity on a variety of neural cells and includes prevention of cell death and induction of neuronal differentiation. Campana and Myers demonstrated the production of Epo and Epo receptors in cells of the peripheral nerve system [88]. This group proposed a model in which Schwann cells are a major target for Epo in injured peripheral nerves, and stated that Epo-receptor-induced cell signaling and Schwann cell proliferation may protect injured peripheral nerves and promote regeneration [89]. Allaf et al. were the first to show expression of Epo receptor in neuronal cell bodies of the major pelvic ganglion, penile nerves, and endothelial cells in the penis, and more recently, in the periprostatic neurovascular bundles [90, 91]. They investigated the potential benefits of Epo on erectile recovery following cavernous nerve injury, and found significantly greater maximal intracavernous pressure area under the curve normalized to mean arterial pressure in EPO treated versus saline treated animals. Morphologically, electron microscopy revealed significant improvement in axonal regeneration in rhEPO treated animals 14 days following injury compared to controls [90].

### 5.4. Stem cell therapy

Although the number is still limited, some studies on stem cell therapy for erectile dysfunction have recently been conducted. Human mesenchymal stem cells (B10) and neural crest stem cells (K10), transplanted via a retroviral vector into the rat corpus cavernosum, have been shown to posess the ability to differentiate into smooth muscle cells or endothelial cells [92, 93]. Damage to smooth muscle and endothelium plays a major role in the development of veno-occlusive dysfunction and ED following radical prostatectomy. Therefore B10 and K10 cells may be an ideal cell source for reconstructing endothelial and smooth muscle cells in the corpus cavernosum in cell therapy for neurogenic ED. Bochinski et al. used embryonic stem cells that differentiated along the neuronal cell line, which they injected into the corpus cavernosum of a rat bilateral CN crush injury model. They measured a markedly increased ICP upon electrical CN stimulation compared to controls, and a greater degree of neuroregeneration of n-NOS containing nerve fibers in the stem cell treatment group [94]. Bella et al. were able to demonstrate in vitro neurite outgrowth from the major pelvic ganglion of the rat induced by adult adipose tissue-derived stem cells, which were not induced towards a specific neuronal cell line prior to administration [95].

## 6. CONCLUSION

Although nerve-sparing techniques are rapidly emerging, ED following radical prostatectomy remains a major problem for the prostate cancer patient and his urologist. Disease-free survival remains the primary goal of radical prostatectomy, although since patients are increasingly younger at the time of diagnosis and treatment, more attention is being paid to quality of life issues. The underlying mechanisms leading to ED have been elucidated by various research institutes. These insights have led to the use of intracavernosal vasoactive substances and more recently, oral PDE5-I on a daily basis in patients that develop ED. Currently, no prophylactic treatment is administered at the time of CN injury. The benefits of these therapeutic modalities have been demonstrated in preclinical as well as in clinical studies. More recently, however, the focus of research has been on the effects of neuromodulatory molecules and stem cell therapy. Until now, this new class of agents has only been shown to be effective in reclinical experiments, although FDA approval has been given for the use of nonimmunosupressant immunophilin ligands. Stem cell therapy is however still experimental. The increasing insights in mechanisms behind ED following radical prostatectomy and the identification of novel substances that promote CN recovery may lead to a new spectrum of treatment modalities for ED following radical prostatectomy.
